# Magnetic Resonance Imaging in 50 Captive Non-domestic Felids - Technique and Imaging Diagnoses

**DOI:** 10.3389/fvets.2022.827870

**Published:** 2022-02-08

**Authors:** Silke Hecht, Andrew C. Cushing, Dottie A. Williams-Hagler, Linden E. Craig, William B. Thomas, Kimberly M. Anderson, Edward C. Ramsay, Gordon A. Conklin

**Affiliations:** ^1^Department of Small Animal Clinical Sciences, University of Tennessee, Knoxville, TN, United States; ^2^Department of Biomedical and Diagnostic Sciences, University of Tennessee, Knoxville, TN, United States

**Keywords:** MRI, brain, spine, neurology, tiger, lion, cat, feline

## Abstract

Magnetic resonance imaging (MRI) is the recognized gold standard for diagnostic imaging of the central nervous system in human and veterinary patients. Information on the use of this modality and possible imaging abnormalities in captive non-domestic felids is currently limited to individual case reports or small case series. This retrospective study provides information on technique and imaging findings in a cohort of cases undergoing MRI at an academic Veterinary Medical Center. The University of Tennessee College of Veterinary Medicine MRI database was searched for non-domestic felids undergoing MRI of the brain or spine from 2008 to 2021. Medical record data were recorded, and MRI studies were reviewed. Fifty animals met the inclusion criteria. The most common brain diseases were Chiari-like malformation (*n* = 8) and inflammatory conditions (*n* = 8). Other abnormalities included pituitary lesions (*n* = 5), brain atrophy (*n* = 2), and one each of metabolic and traumatic conditions. Fourteen animals had a normal brain MRI study. The most common spinal abnormality was intervertebral disc disease (*n* = 7). Other disorders included vertebral dysplasia (*n* = 2), presumptive ischemic myelopathy (*n* = 1), subdural ossification causing spinal cord compression (*n* = 1), and multiple myeloma (*n* = 1). Spinal cord swelling of undetermined cause was suspected in two animals, and seven patients had a normal MRI study of the spine. MRI is a valuable tool in the diagnostic workup of non-domestic felids with presumptive neurologic disease.

## Introduction

With the exception of Chiari-like malformation and degenerative intervertebral disc disease, publications on diseases of the central nervous system in non-domestic felids are generally scarce and mostly consist of post mortem data, small case series, and individual case reports. An ante-mortem diagnosis of brain or spinal disease typically requires some form of diagnostic imaging. Radiography is readily accessible and may provide helpful information especially in the evaluation of osseous structures (skull and vertebrae), e.g., in cases of trauma (1).

Computed tomography (CT) is increasingly available in zoological institutions and sanctuaries and has the advantage of rapid image acquisition resulting in short anesthesia times (2). CT is the gold standard for the evaluation of bony structures but has limitations in soft tissue imaging. Myelography involving the injection of contrast medium into the subarachnoid space can be combined either with radiography or CT (3–6). It provides improved delineation of the subarachnoid space and is an excellent test for the diagnosis of compressive myelopathies such as intervertebral disc extrusion. However, it is technically challenging and associated with a possible risk of adverse reaction to contrast medium injection.

Magnetic resonance imaging (MRI) is the recognized gold standard for diagnostic imaging of the central nervous system in human and veterinary patients. Information on the use of this modality and possible imaging abnormalities in captive non-domestic felids is currently limited to individual case reports or small case series. MRI findings reported in a Bengal tiger with hypoxic encephalopathy included bilaterally symmetric T2 hyperintense brain lesions with restricted diffusion and evidence of intralesional hemorrhage (7). MRI findings in 4 lion cubs diagnosed with calvarial hyperostosis/Chiari-like malformation secondary to suspected hypovitaminosis A included a small caudal fossa with compression of the cerebellum from dorsally and caudally by the thickened osseous tentorium and/or occipital bone, foramen magnum herniation of the cerebellum, lateral ventriculomegaly with compression of the third and fourth ventricles, effacement of the CSF signal from the cerebellar sulci, cervical syringomyelia and in some cases concurrent abnormal conformation of the atlas and axis resulting in spinal cord compression (8). Similar abnormalities are reported in another young lion and a bobcat with this condition (9, 10). Leukoencephalopathy in cheetahs (*Acinonyx jubatus*) has been reported to cause bilaterally symmetric white matter changes best seen on T2-weighted images (11). The MRI examination in a tiger with suspected *Clostridium perfringens* neurotoxicosis did not reveal any abnormalities other than an incidental empty sella (12). MRI findings in a tiger with noncompressive segmental myelopathy suspected to represent a fibrocartilaginous embolism included regional T2 hyperintensity of the spinal cord and decrease in normal T2 hyperintensity of regional intervertebral discs associated with mild noncompressive intervertebral disc protrusions (13). Multifocal bone lesions were reported in a tiger ultimately diagnosed with multiple myeloma (14). Information on MRI technique and findings in a larger cohort of non-domestic felids is lacking to date.

The purpose of this retrospective study was to provide information on technique and imaging findings in captive non-domestic felids undergoing MRI at an academic Veterinary Medical Center.

## Materials and Methods

The University of Tennessee College of Veterinary Medicine MRI database was searched for captive non-domestic felids undergoing MRI of the brain and/or spine from 2008 to 2021. Inclusion criteria were a complete or partial ante- or postmortem MRI examination in felids suspected to have neurologic disease. Normal animals scanned in the frame of research projects or for comparison purposes were excluded. Animals included in a large retrospective case series on brain lesions in captive non-domestic felids identified on autopsy (15) were included, as diagnostic imaging was not part of this prior publication. Animals previously published as case reports were also included (10, 14).

Medical record data were recorded by an ACVIM neurology resident (DW) supervised by 2 ACVIM-Neurology diplomates (KA, WT), 2 ACZM diplomates (AC, ER), and an ACVR/ECVDI diplomate (SH) and included species, age, sex, weight, pertinent medical history, and, if available, information on cerebrospinal fluid (CSF) analysis, results of additional tests, final clinical diagnosis, and autopsy results. Additional information on clinical management and outcome was collected if possible by contacting the animals' caretakers.

All MRI studies were reviewed by an ACVR/ECVDI board-certified radiologist (SH). The scan site and imaging diagnosis were recorded. Additional information captured included the MRI system used, if contrast medium was administered, and if any adverse effects to contrast medium administration were noted.

For lions (*Panthera leo*) undergoing MRI examination of the brain, the foramen magnum height/skull width (FMH/SW) ratio was calculated as previously described (16).

The MRI diagnosis was established based on imaging findings and available information on signalment, history and clinical presentation. The final clinical diagnosis was based on the MRI diagnosis and results of additional tests, if available.

The descriptive statistical analysis was performed by the first author. Quantitative data are presented as count numbers or percentages. Quantitative data are presented as the median and range.

## Results

Detailed individual patient data are provided in Appendix 1.

### Animals

Fifty cats met the inclusion criteria. Forty-eight patients were scanned alive. A limited postmortem examination was performed in two animals. Ten animals were included in prior publications [(10, 14, 15); see Appendix 1].

Eighteen tigers (*Panthera tigris*), 11 lions (*Panthera leo*), 4 leopards (*Panthera pardus*), 3 each of bobcats (*Lynx rufus*), servals (*Leptailurus serval*) and snow leopards (*Pantera uncia*), 2 each of cheetahs (*Acinonyx jubatus*) and caracals (*Caracal caracal*), and 1 each of clouded leopard (*Neofelis nebulosa*), cougar (*Puma concolor*), liger (*Panthera leo x Panthera tigris*), and lynx (*Lynx canadensis*) met inclusion criteria. The majority of cats (41) belonged to a single large cat sanctuary. One lion, one cheetah and one tiger belonged to zoological institutions, and the three servals, one caracal and one lion were privately owned.

There were 19 female (13 intact, 6 spayed) and 31 male (25 intact, 6 neutered) cats. The median age was 8 years (range, 0.5–18 years); the age of one leopard was unknown. The median weight was 105 kg (range, 2.36–280 kg); the weight of one tiger was not recorded.

### MRI Scan Regions And Technique

**Figure 1:**
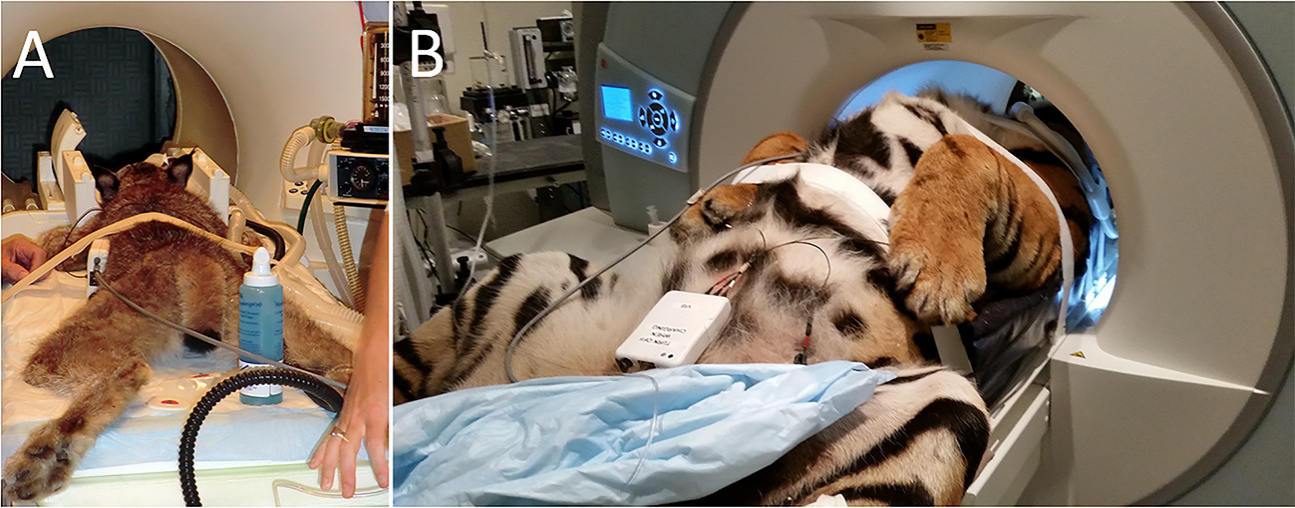
Examples of patient positioning for the MRI examination. **(A)** A lynx being positioned in sternal recumbency for MRI examination of the head. **(B)** A tiger being positioned in dorsal recumbency for MRI examination of the head and cervical spine.

MRI scan regions in live animals included the brain (*n* = 28); brain and cervical spine (*n* = 9); cervical spine (*n* = 1); thoracolumbar spine (*n* = 3); lumbosacral spine (*n* = 4); cervical and thoracic/thoracolumbar spine (*n* = 2); and brain, cervical and thoracolumbar spine (*n* = 1). Scan regions post mortem included the brain (*n* = 1) and brain and cervical spine (*n* = 1). Eighteen scans were performed using a 1.0T MRI system (MAGNETOM Harmony^TM^, Siemens Medical Solutions, Malvern, PA), and 32 scans were performed using a 1.5T MRI system (MAGNETOM Espree^TM^, Siemens Medical Solutions, Malvern, PA). Live animals were scanned under general anesthesia. Smaller felids undergoing brain MRI were positioned either in sternal (Figure 1A) or dorsal recumbency. Larger animals undergoing brain MRI and animals undergoing MRI examination of the spine were positioned in dorsal recumbency (Figure 1B). Coil selection and MRI protocols varied between patients and scan areas. A brain and/or neck coil was generally used for imaging of the head. Imaging of the cervical spine was performed using a neck coil. Spine coils were used for imaging of the thoracolumbar and lumbosacral spine.

MRI protocols varied between patients and were limited in some instances based on clinical suspicion, imaging findings, and number of scan areas. A full MRI examination of the brain generally included sagittal T2-weighted (T2-W), transverse T2-W, T1-weighted (T1-W), T2-FLAIR (fluid attenuated inversion recovery), T2^*^-W gradient recalled echo (GRE), and post contrast T1-W images with or without fat saturation (FatSat) in 3 planes. Additional sequences acquired on a case-by-case basis included diffusion weighted imaging (DWI), transverse proton density (PD) weighted images, transverse “SPACE” (“Sampling Perfection with Application optimized Contrasts using different flip angle Evolution”), and transverse post contrast “VIBE” (“Volume Interpolated Breathhold Examination”) images. A full MRI examination of the spine generally included a dorsal STIR (short tau inversion recovery), sagittal T2-W, T1-W, STIR and “HASTE” (“Half-Fourier Acquisition Single-shot Turbo spin Echo imaging”; MR myelogram), and transverse T1-W and T2-W images. Additional sequences acquired on a case-by-case basis included a transverse SPACE and post contrast T1-W FatSat images in 1-3 planes. The scan parameters used for small cats were identical to those used for similar sized canine patients. Protocols were modified for larger cats. Sample protocols for imaging the brain and spine of a large cat on a 1 and 1.5T MRI system are provided in Appendix 2.

### MRI Findings – Brain

A total of 39 felids had a partial or complete MR examination of the brain.

#### Congenital/Developmental Anomalies

**Figure 2:**
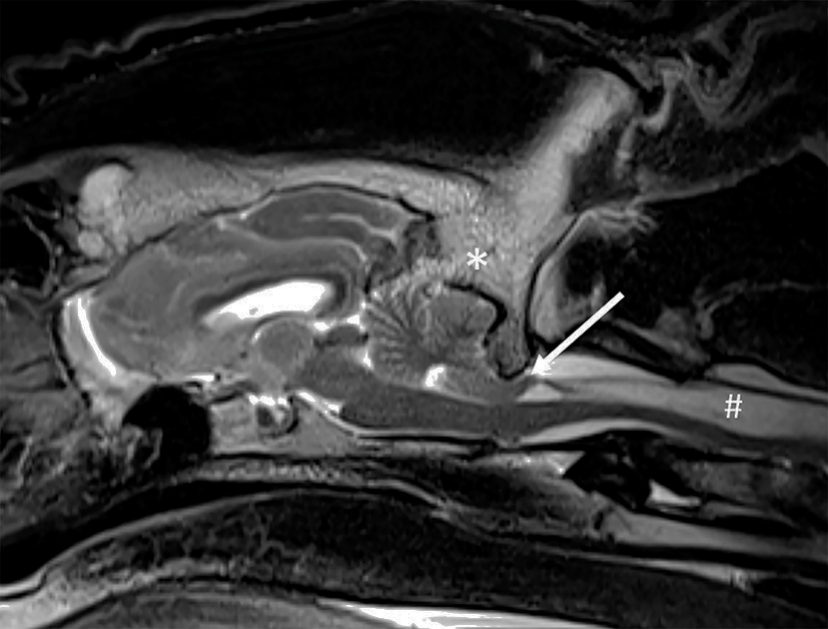
Chiari-like malformation in a lion. On this T2-W sagittal image there is thickening of the occipital bone and osseous tentorium of the cerebellum (*), crowding of the caudal fossa, cerebellar compression and herniation (arrow), and marked syringomyelia of the cranial cervical spinal cord (#).

Eight animals were included in this category. Six animals (4 lions, 1 tiger and 1 bobcat, ranging in age from 0.8–17 years) had an MRI diagnosis of Chiari-like malformation (caudal occipital malformation syndrome/calvarial hyperostosis) believed to be responsible for the clinical signs. Another lion was diagnosed with calvarial hyperostosis but also tested positive for toxoplasmosis. The clinical significance of calvarial hyperostosis was therefore undetermined. One additional lion had a diagnosis of “probable occipital hyperostosis” but had only mild clinical signs at presentation and was reported to be neurologically normal 8 years after the MRI study. Imaging abnormalities in affected cats included thickening of the occipital bone and osseous tentorium of the cerebellum, crowding of the caudal fossa, variable degree cerebellar compression and herniation, and syringomyelia of the cranial cervical spinal cord (Figure 2). Two animals were treated surgically (foramen magnum decompression); the other animals were managed conservatively. Of the cats treated surgically, one was discharged and lost to follow-up. The other, a 3-year-old bobcat, did not improve following surgery. Repeat MRI in 3 months later showed evidence of cerebellar herniation and syringomyelia, and the patient was euthanized. The imaging abnormalities were confirmed on autopsy. Of the animals treated conservatively, two were euthanized 4 and 18 months following MRI, respectively. Abnormalities consistent with Chiari like malformation/calvarial hyperostosis were confirmed postmortem. Additional intracranial abnormalities in the cat with the longer timeframe between MRI and euthanasia included an infarct of the left caudate nucleus and a meningioma in the fourth ventricle. Neither of these lesions was evident on the MRI study at the initial interpretation or in hindsight, and it is likely that they were not present at the time of the study. One animal died and no autopsy was performed. Three felids managed conservatively are alive 8 years after MRI. One is neurologically normal, one exhibits occasional neurologic deficits, and one is neurologically unchanged from the time of presentation.

#### Inflammatory Conditions

Inflammatory conditions of the intracranial and/or other structures of the head were diagnosed or suspected in eight patients.

**Figure 3:**
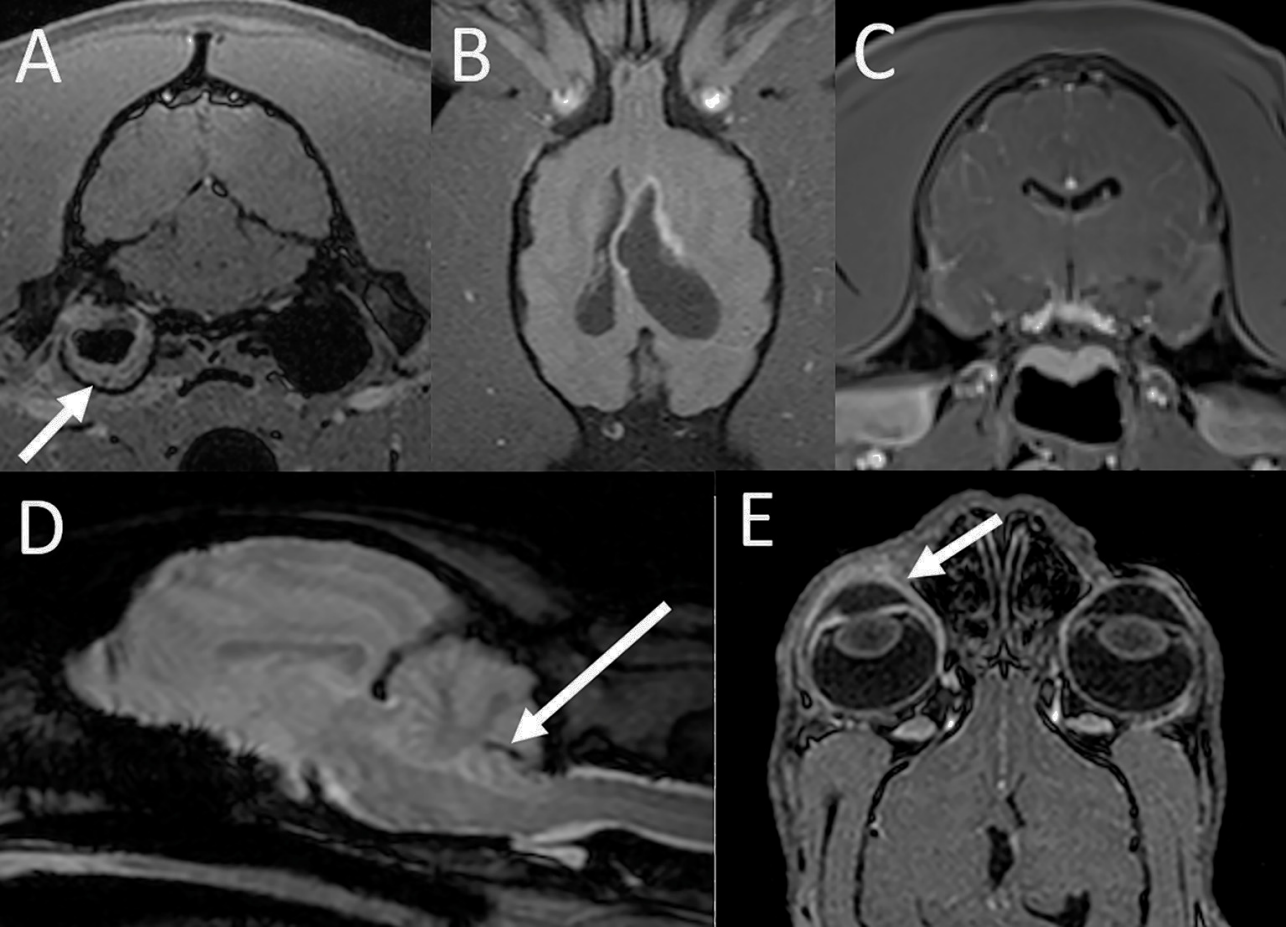
MRI findings with inflammatory conditions. **(A)** Otitis media and interna in a tiger. The transverse post contrast T1-W GRE image with FatSat (“VIBE”) shows soft tissue material along the periphery of the right tympanic bulla (arrow). There is also mild contrast enhancement of the soft tissues adjacent to the bulla consistent with regional inflammation. **(B)** Intracranial blastomycosis in a tiger. On the dorsal T1-W FatSat image there is asymmetric dilatation of the lateral ventricles with marked contrast enhancement of the ventricular lining especially along the rostral horn of the left lateral ventricle. **(C)** Lymphoplasmacytic meningoencephalitis in a tiger. On the transverse post contrast T1-W GRE image with FatSat (“VIBE”) there is evidence of marked mostly leptomeningeal contrast enhancement. **(D)** Parasitic meningoencephalitis in a bobcat. A linear susceptibility artifact is associated with the cerebellum (arrow), consistent with a hemorrhagic migration tract. **(E)** Corneal ulcer, keratitis and uveitis in a lynx. The dorsal post contrast T1-W GRE image with FatSat (“VIBE”) shows contrast enhancement of the right cornea and uvea (arrow) compared to the left.

Three animals had a diagnosis of otitis media, with or without otitis interna. A 4-year-old snow leopard presented with recent vision loss, decreased mentation, and decreased response to stimuli had evidence of bilateral otitis media and questionable meningeal enhancement suggesting possible intracranial extension. The brain parenchyma was normal. On autopsy 3 weeks following the MRI study, the patient had meningoencephalitis with vasculitis, possibly of viral cause; likely unrelated to the MR diagnosis of otitis. A second patient, a 13-year-old tiger previously diagnosed with chronic otitis media based on CT examination, underwent MRI due to the recent onset of seizures. The MRI examination confirmed otitis media and interna with extension into the adjacent soft tissues of the caudal head but without evidence of involvement of the intracranial structures (Figure 3A). Regional cellulitis without evidence of extension of the inflammatory process into the brain and/or spinal cord was confirmed on autopsy. The reason for seizures was not identified. A 10-year-old tiger presented with a head tilt for 3 weeks. Otitis media and interna were diagnosed based on MRI examination, and the patient was medically managed. Based on verbal follow-up, the animal is alive 11 years after the MRI examination and is mostly normal with an occasional head tilt.

A 17-year-old tiger that had been treated for blastomycosis 18 months prior presented for neurologic deficits and was found to have asymmetric hydrocephalus associated with marked contrast enhancement of the ventricular lining (Figure 3B). CSF analysis revealed mild-to-moderate mixed cell (predominantly neutrophilic) pleocytosis. Intracranial blastomycosis was confirmed on postmortem examination.

A 1-year-old tiger was presented with acute onset seizures, ataxia, and nystagmus. MRI examination revealed ill-defined and indistinct multifocal intra-axial brain lesions as well as marked meningeal enhancement especially affecting the leptomeninges (Figure 3C). Based on these abnormalities and results of the CSF analysis (marked neutrophilic pleocytosis) a diagnosis of meningoencephalitis was made. Empirical treatment was unsuccessful. Postmortem examination yielded a diagnosis of lymphoplasmacytic meningoencephalitis, possibly of viral etiology. There was also acute cerebellar herniation with multifocal hemorrhage and encephalomalacia not present on the MRI examination performed several days prior to euthanasia.

A 12-year-old bobcat with head shaking, ataxia, and hyporeflexia of one-week duration was found to have a tract-like cerebellar hemorrhage on MRI, suggestive of possible parasite migration (Figure 3D). The CSF analysis revealed mild mononuclear leukocytosis and reactivity as well as evidence of prior hemorrhage. The patient was medically managed and had fairly static clinical signs for a period of 5 months but then suddenly deteriorated and died. On autopsy there were multiple cerebellar linear tracts of hemosiderin with reactive glial cells, suggestive of prior parasite migration. There was also evidence of granulomatous inflammation and cerebral gliosis.

A 5-year-old bobcat was presented with a variety of clinical and neurologic deficits consistent with cerebellar and/or brainstem neuroanatomic localization. The MRI examination of the brain was normal, however, based on CSF analysis the patient was diagnosed with neutrophilic meningoencephalitis. No infectious organisms were identified. The animal was successfully medically managed and remained normal for more than 10 years following the MRI examination until its death without recurrence of the previous clinical signs.

A 6-year-old lynx presented for disorientation, opisthotonos, ataxia and protrusion of the eyelid. The MRI examination did not show any abnormalities of the intracranial structures; however, contrast enhancement of the cornea and uvea was noted (Figure 3E). A subsequent ophthalmologic exam under general anesthesia revealed a corneal ulcer, and a diagnosis of corneal ulcer, keratitis and uveitis was made. A reason for neurologic deficits at time of presentation was not identified. The patient was treated with topical medications for the corneal ulcer and empirically for possible concurrent/underlying CNS disease and recovered with supportive care. This animal was euthanized as a geriatric cat for unrelated causes 9 years after the MRI examination with no neurologic deficits reported at the time of euthanasia.

#### Metabolic Encephalopathies

**Figure 4:**
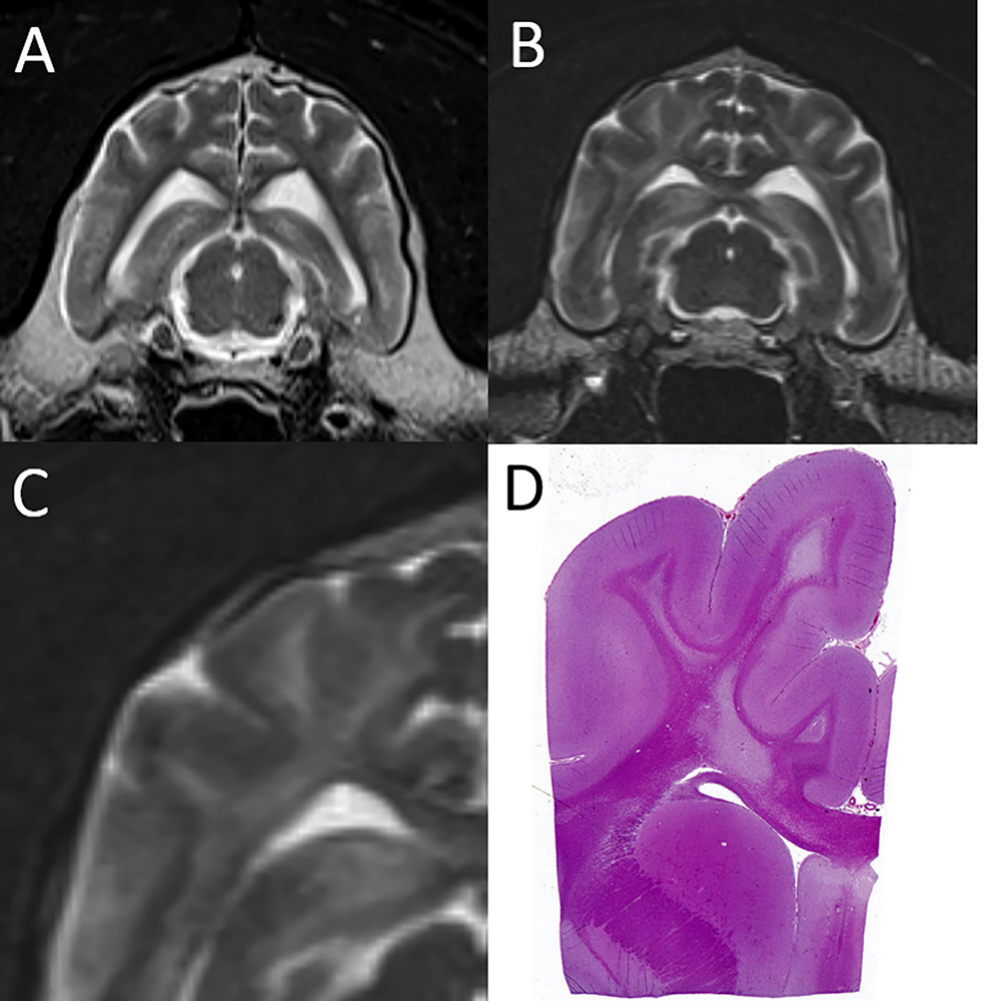
Leukoencephalopathy in a snow leopard. **(A)** On this normal transverse T2-W image of the brain in a different leopard, the centrally located finger-like white matter tracts are hypointese to gray matter. **(B)** In the affected leopard the white matter tracts are diffusely T2 hyperintense. **(C,D)** Magnification of the T2-W MRI image paired with the corresponding histopathology image, which shows severe pallor and loss of cerebral white matter. Courtesy of Dr. Mee-Ja Sula.

A 16-year-old snow leopard presented with acute onset of ataxia and blindness. Following slow recovery from anesthesia he sustained a seizure during recovery and was euthanized. A limited MRI examination of the brain was performed postmortem. There was marked diffuse bilaterally symmetric white matter disease (Figure 4), consistent with a metabolic/toxic/nutritional or degenerative leukoencephalopathy. The MRI diagnosis was confirmed on postmortem examination.

#### Trauma

**Figure 5:**
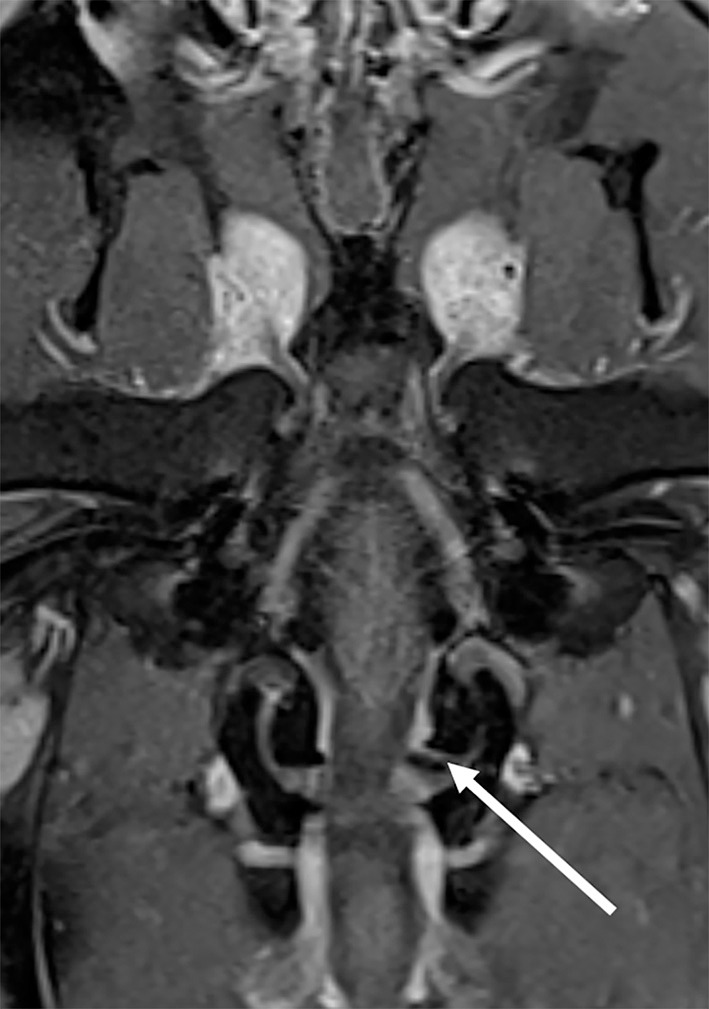
Fracture of the left occipital condyle in a lion (arrow) which was best seen on this dorsal T1-W post contrast FatSat image. The caudal skull was only partially or not included in the scan field of view on most sequences, and the lesion was missed during the initial image interpretation.

A 15-year-old lion developed ataxia in both front limbs approximately 3 weeks prior to presentation which was attributed to complications of a recent declaw repair and was managed medically. Immediately prior to presentation, the animal was found in sternal recumbency unable to sit or stand up. The MRI examination revealed a fracture of the occipital condyle that was subsequently confirmed on autopsy (Figure 5). The lesion was only partially included in the scan field of view and was missed during the initial interpretation of the MRI study.

#### Degenerative Conditions

**Figure 6:**
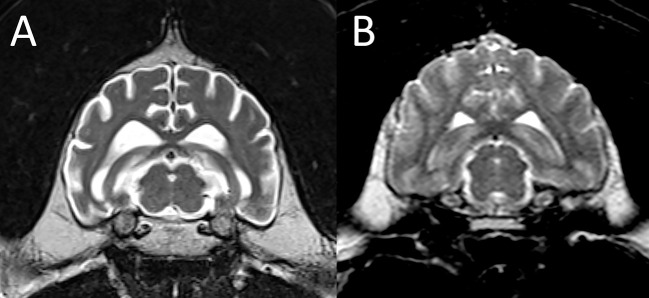
Brain atrophy. T2-W transverse images of an affected 18-year-old tiger **(A)** compared to a 13-year-old control animal of the same species **(B)**. Note generalized increase in ventricular size and widening of the cerebral sulci due to diffuse brain parenchymal loss in the affected cat **(A)**.

Marked diffuse brain atrophy (Figure 6) was documented in an 18-year-old tiger presented with ataxia and dull mentation which progressed to obtundation, paresis, and blindness. The changes were considered consistent with age/cognitive dysfunction. Short-term medical management was attempted with little changes in clinical signs. The animal was euthanized 2 weeks after MRI. On autopsy, there was generalized gliosis and satellitosis.

Lesser degree brain atrophy was also seen in a 15-year-old cheetah with staggering, moving slowly, falling, stiff gait, somnolence and disorientation. The animal was euthanized 2 years following the MRI examination for progressive likely age-related deficits. The brain was normal on gross inspection postmortem; histopathological examination was not performed.

#### Other Abnormalities

Pituitary abnormalities were noted in five animals.

**Figure 7:**
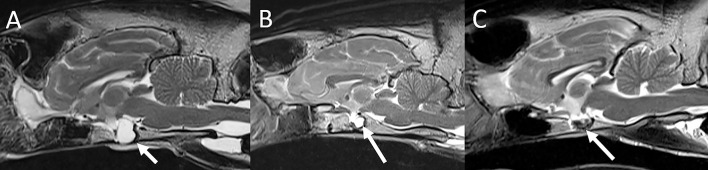
Pituitary abnormalities. Sagittal T2-W images of the brain showing **(A)** a large cyst-like lesion (cystadenoma) associated with the pituitary fossa in a cougar, **(B)** an empty sella in a tiger, and **(C)** a mildly nodular and heterogeneous pituitary gland (mild chronic multifocal nodular pituitary hyperplasia) in a leopard (arrows).

A large cyst-like lesion was noted associated with the pituitary fossa in a 14-year-old cougar (Figure 7A) presenting for behavioral changes, blindness, and anisocoria. Autopsy performed 1 month after MRI found a pituitary cystadenoma believed to be associated with the ophthalmologic abnormalities.

An empty sella was identified in a 5-year-old tiger presented with a suspected focal seizure and was considered an incidental finding unrelated to the clinical signs (Figure 7B). The same animal also had asymmetry of the frontal sinuses, likely congenital. An empty sella was also noted as an incidental finding in an 8-year-old caracal suspected to be affected by possible spinal meningitis (see below). No pituitary abnormalities were documented on autopsy in this patient.

A 12-year-old leopard with obtundation, ataxia and anisocoria had a mildly nodular and heterogeneous pituitary gland (Figure 7C) confirmed to represent mild chronic multifocal nodular pituitary hyperplasia on autopsy. The association of the pituitary abnormalities with the neurologic abnormalities remained undetermined. The same cat had a diagnosis of cervical intervertebral disc disease (see below).

Finally, a 10-year-old tiger presenting with vague neurologic signs including ataxia and subsequently diagnosed with intervertebral disc disease (see below) had an enlarged and cystic pituitary gland with a suspected small focus of hemorrhage. A limited postmortem examination was performed and no pituitary abnormalities were reported.

#### Normal MRI Studies

In addition to the bobcat with neutrophilic meningoencephalitis mentioned above, 14 felids had a normal MRI examination of the brain. In one of these, a 0.5-year-old caracal, a diagnosis of presumptive idiopathic epilepsy was made, and the patient's seizures were controlled with medical management. For the 13 remaining animals, a reason for the presenting signs was either found in the spine (*n* = 4; see section on spine below), outside the CNS (*n* = 2), or remained undetermined (*n* = 7).

#### Foramen Magnum Height/Skull Width (FMH/SW) Ratio in Lions

The FMH/SW ratio was determined in lions as previously described (16). The median FMH/SW ratio in lions diagnosed with Chiari-like malformation was 0.06 (range, 0.056–0.093). The median FMH/SW ratio in lions with other diagnoses was 0.069 (range, 0.055–0.09).

### MRI Findings – Spine

A total of 21 felids had a partial or complete MRI examination of the spine.

#### Intervertebral Disc Disease

**Figure 8:**
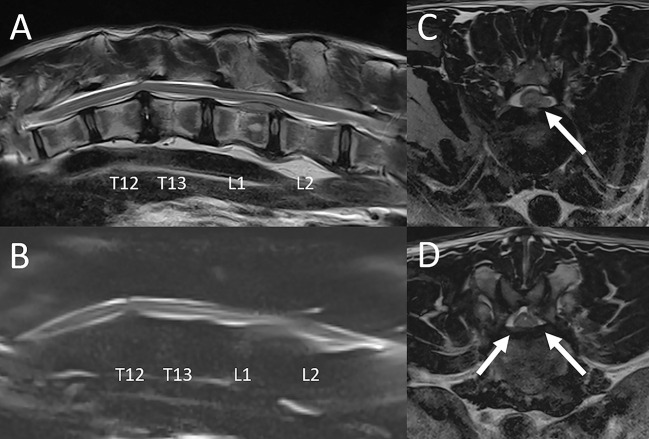
Intervertebral disc disease in a tiger. Sagittal T2-W image **(A)**, sagittal MR myelogram [HASTE; **(B)**], and transverse T2-W images at L1-2 **(C)** and T12-13 **(D)**. There is variable degree decrease of normal T2 hyperintensity of all intervertebral discs included in the scan field of view. **(A)** At L1-2 there is a moderate amount of intermediate intensity extradural material associated with the vertebral canal, extending from the level of the L1-2 intervertebral disc space cranially to mid-body L1. There is intramedullary hyperintensity of the spinal cord over the length of the lesion and extending to mid-body L2. The T12-13 intervertebral disc and to a lesser degree the T13-L1 intervertebral disc protrude into the ventral aspect of the vertebral canal. **(B)** On the MR myelogram there is complete circumferential attenuation of the subarachnoid space over the length of L1 and cranial L2. The ventral subarachnoid space at T12-13 is dorsally deviated but remains visible. **(C)** Herniated disc material +/- associated hemorrhage is located in the left ventral aspect of the vertebral canal at L1-2 (arrow), causing moderate spinal cord compression. **(D)** At T12-13 herniated disc material is located in the ventral aspect of the vertebral canal and is contiguous with the annulus of the intervertebral disc (arrows). There is only mild spinal cord compression from ventral at this level. The findings are consistent with acute intervertebral disc extrusion +/- associated extradural hemorrhage at L1-2 causing moderate spinal cord compression from left ventral. Spinal cord hyperintensity at this level is consistent with edema. There are concurrent chronic intervertebral disc protrusions, most notably at T12-13, not causing significant spinal cord compression.

The evaluation and classification of intervertebral disc disease was performed using previously published criteria (17, 18). A diagnosis of clinically significant intervertebral disc disease was made in seven felids. Four animals (2 lions and 2 tigers ranging in age from 7–17 years) had evidence of intervertebral disc extrusion localized to the cervical (*n* = 2) or to the lumbar spine (*n* = 2) (Figure 8). All animals had concurrent evidence of multifocal spinal degenerative changes including concurrent multifocal intervertebral disc degeneration. The diagnosis was confirmed either at surgery or autopsy in all cases. One tiger underwent surgery (right hemilaminectomy at C4-5) but had to be euthanized 5 weeks following MRI due to lack of clinical improvement. One lion was euthanized immediately following the MRI study. Two animals were treated conservatively but were euthanized 2.5 and 3.5 months following MRI due to lack of clinical improvement. In three animals (two tigers and one leopard ranging in age from 10–14 years) there was evidence of chronic intervertebral disc disease with protrusion causing variable degree spinal cord compression in the cervical spine (*n* = 2) or lumbosacral spine (*n* = 1). All three animals were medically managed. Two were euthanized 1 week and 4 months following MRI, respectively, due to lack of clinical improvement. One leopard remained stable neurologically and died of unrelated causes 5 years following the MRI examination.

#### Congenital/Developmental Anomalies

Two young animals, a 0.8-year-old female leopard and a 0.75-year-old male liger, were diagnosed with congenital vertebral anomalies ultimately confirmed to be vertebral dysplasia.

The leopard presented with an acute onset of neurologic deficits after a fall. A possible episode of ataxia and lethargy may have occurred the week prior. MRI findings included foreshortening and abnormal shape of the C2-C5 vertebral bodies, irregular endplate margination of these vertebrae, loss of normal T2 hyperintensity of the C2-C5 intervertebral discs, and flattening and compression of the spinal cord. The imaging findings were consistent with congenital vertebral dysplasia. There was also the impression of diffuse cervicothoracic spinal cord swelling and meningeal enhancement, possibly suggesting concurrent meningomyelitis. This animal was euthanized 6 weeks following MRI due to progressive decline in mentation and mobility. Cervical vertebral dysplasia with spinal cord compression was confirmed on autopsy.

**Figure 9:**
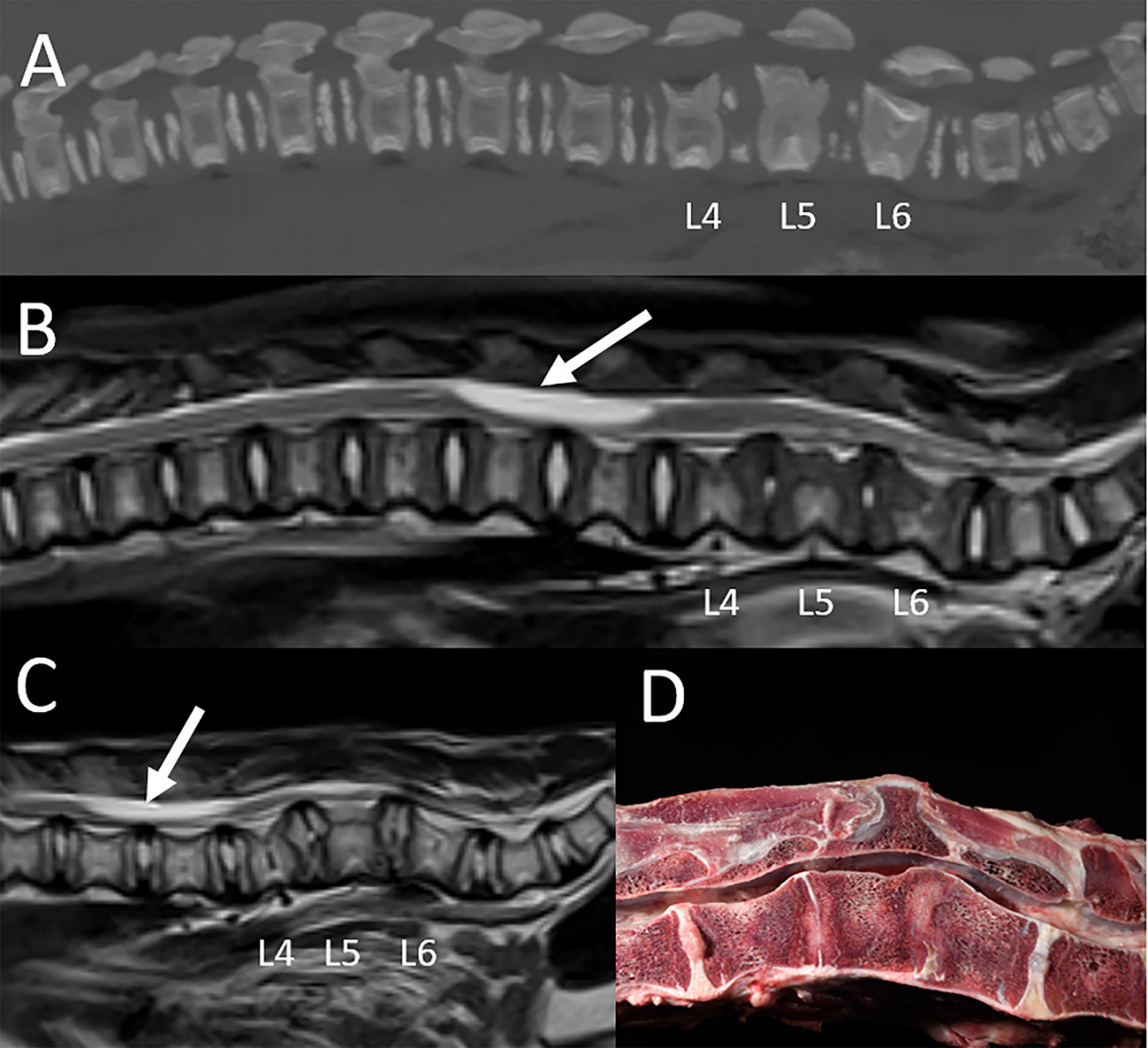
Vertebral dysplasia in a liger. MRI and CT were performed at 0.75 years of age, and the MRI examination was repeated at 1.25 years of age. **(A)** Sagittal reconstruction of the CT images, **(B)** sagittal T2-W image obtained at the first MRI examination, **(C)** sagittal T2-W image obtained at the repeat MRI examination, and **(D)** corresponding gross pathology image. Imaging findings include foreshortening and abnormal shape of the L4 through L6 lumbar vertebrae, with decrease in intervertebral disc space width, decrease in size and normal T2 hyperintense signal of the intervertebral discs, dorsal protrusion of the intervertebral discs, lumbar kyphosis, and spinal cord compression. A large cyst-like intramedullary lesion was identified, most consistent with a syrinx with other cystic conditions not excluded (arrow). Autopsy **(D)** confirmed vertebral dysplasia with severe syringohydromyelia.

The liger presented with chronic paraparesis and proprioceptive ataxia of the pelvic limbs. On neurologic examination, he also failed to demonstrate deep pain perception in the pelvic limbs and tail. This animal was reported to have a sibling with similar clinical signs. MRI findings included foreshortening and abnormal shape of the L4 through L6 lumbar vertebrae, with decrease in intervertebral disc space width, decrease in the normal T2 hyperintense signal of the intervertebral discs, dorsal protrusion of the intervertebral discs, lumbar kyphosis, and spinal cord compression (Figure 9). Computed tomography of the lumbar spine was performed for further evaluation of the osseous abnormalities and yielded similar results. There was also a large cystic lesion associated with the dorsal aspect of the spinal cord over the length of L2-3, most consistent with a syrinx. A concurrent malformation with spinal cord compression was noted at the atlantoaxial junction. A repeat MRI study was performed 6 months later showing similar to progressive imaging abnormalities. He was treated with weekly in-house physical therapy with the goal of slowing progression of clinical signs. The animal was euthanized 14 months after the second MRI study due to a slow progression of clinical signs. On autopsy there was vertebral dysplasia with severe syringomyelia. Additional abnormalities on post mortem examination included intracranial abnormalities (*sella turcica* and *tentorium cerebelli* dysplasia).

#### Neoplasia

**Figure 10:**
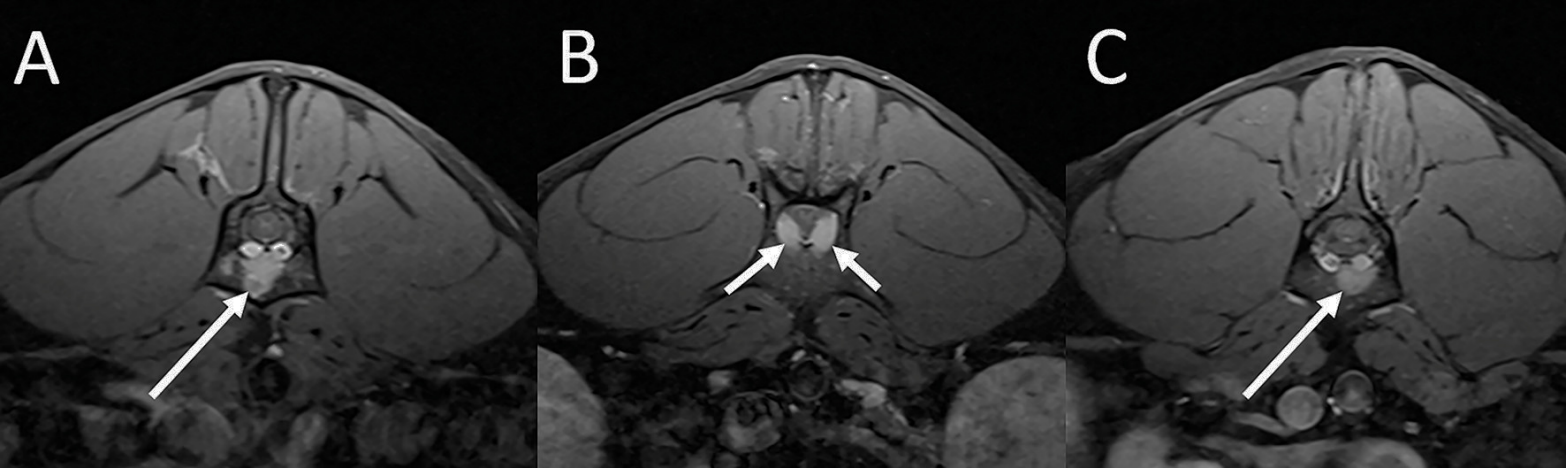
Multiple myeloma in a tiger. Transverse post contrast T1-W FatSat images at the level of L2 **(A)**, L3 **(B)** and L4 **(C)**. There are strongly contrast enhancing polyostotic lesions centered on the medullary cavities of the affected vertebrae, and for the most part exhibit cortical sparing (arrows). The lesion at L3 extends into the vertebral canal, with 2 seemingly separate extradural masses within the left ventral and right ventral aspect of the vertebral canal resulting in marked bilateral ventrolateral spinal cord compression.

A 16-year-old tiger was presented with a two-week history of progressive paraparesis. MRI examination of the lumbosacral spine showed evidence of multifocal vertebral lesions (involving at least L2-L4). The lesions were T1, T2 and STIR hyperintense, strongly contrast enhancing, centered on the medullary cavities of the affected vertebrae, and for the most part exhibited cortical sparing (Figure 10). The lesion at L3 had extension into the vertebral canal, with 2 seemingly separate extradural masses within the left ventral and right ventral aspect of the vertebral canal resulting in marked bilateral ventrolateral spinal cord compression. The imaging findings were consistent with multifocal neoplasia, and considering bone marrow centricity and cortical sparing, round cell neoplasia such as multiple myeloma or lymphoma was given primary consideration. Urine and serum protein electrophoresis suggested monoclonal gammopathy, and radiation therapy and chemotherapy therapy were attempted. However, the animal was euthanized 13 days after MRI due to worsening of neurologic deficits, and multiple myeloma was confirmed on autopsy.

#### Vascular Conditions

**Figure 11:**
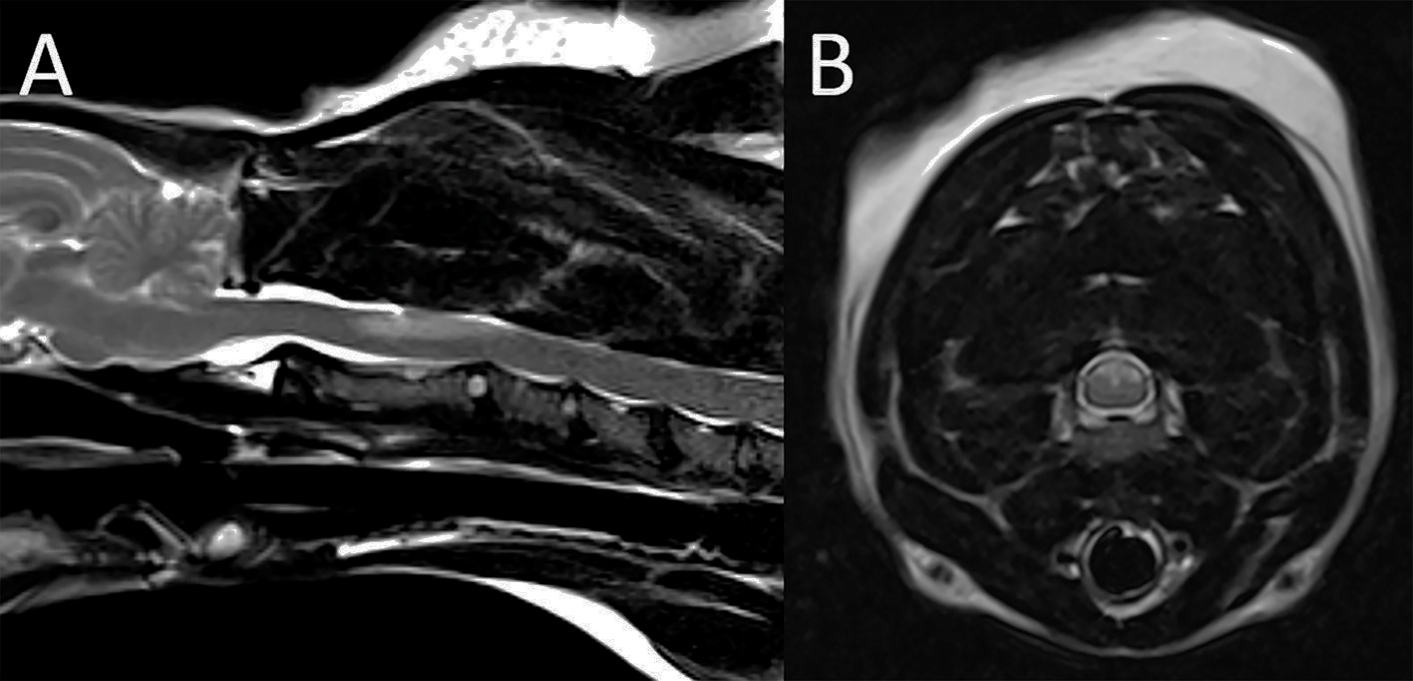
Suspected ischemic event in a leopard. On sagittal **(A)** and transverse **(B)** T2 weighted images there is a T2 moderately hyperintense intramedullary lesion within the dorsal aspect of the spinal cord at the level of C2 which appears symmetric and wedge-shaped on the transverse image. Note also multifocal degenerative intervertebral disc disease.

An 11-year-old clouded leopard presented for ataxia of unknown duration and possible vestibular disease. On MRI there was a T2 moderately hyperintense intramedullary lesion associated with the dorsal aspect of the spinal cord at the level of C2 which was symmetric and wedge-shaped on transverse image (Figure 11). This lesion was considered most consistent with an ischemic event such as feline ischemic myelopathy, even though it was in a more dorsal location than lesions reported with this condition in domestic cats (19, 20). Other intramedullary lesions were not excluded. The animal died 1.5 years following the MRI examination of presumptive renal failure. On autopsy there was unilateral hydrocephalus not evident on the previous imaging study. The spinal cord was normal, supporting the presumptive imaging diagnosis of a vascular accident.

#### Degenerative Conditions

**Figure 12:**
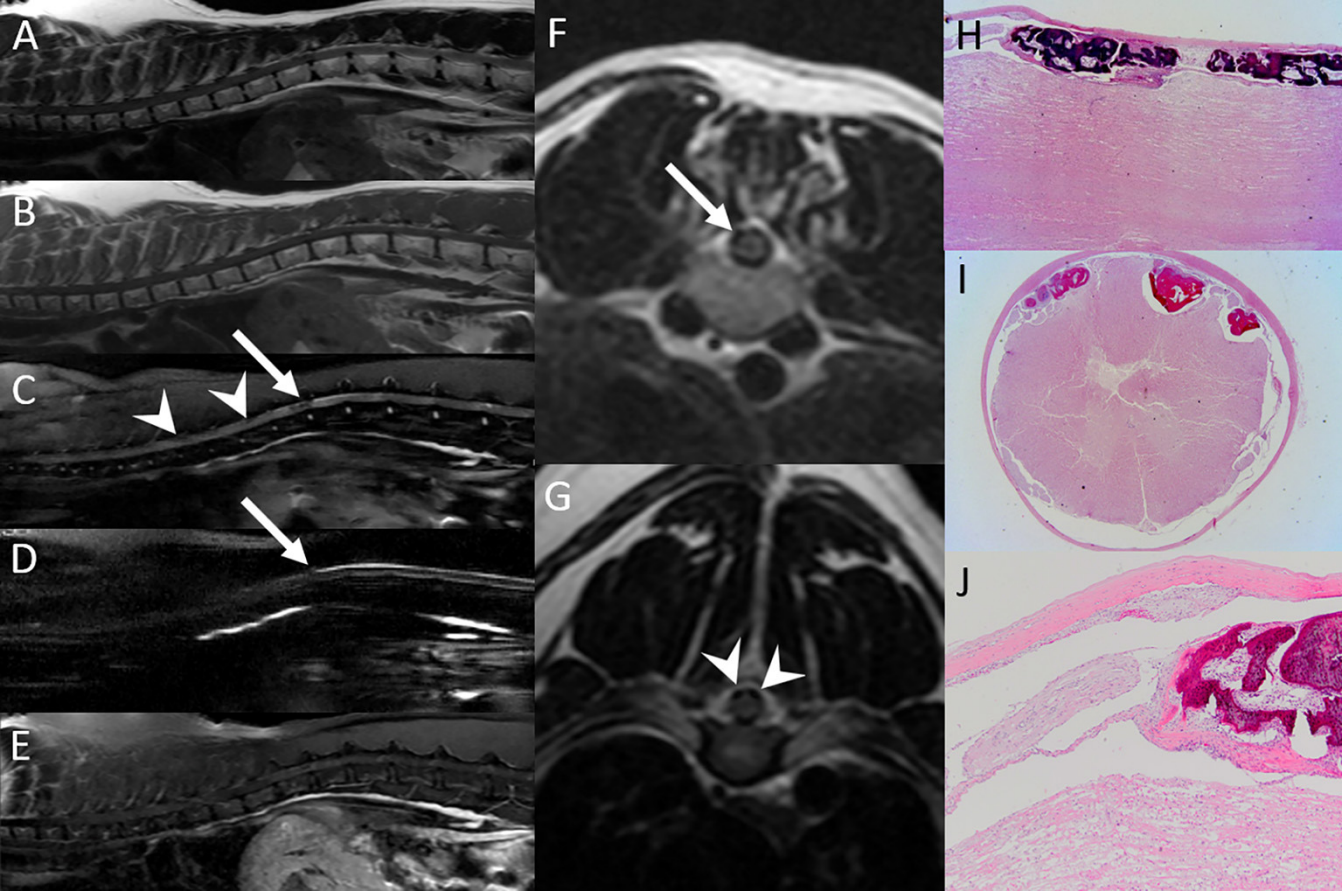
Subdural and subarachnoid ossification resulting in spinal cord compression in a serval. Sagittal T2-W **(A)**,T1-W **(B)**, STIR **(C)**, HASTE **(D)** and post contrast FatSat **(E)** and transverse T2-W images at T11-12 **(F)** and T7-8 **(G)**. Sagittal **(H)** and transverse **(I,J)** pathology images. **(A–G)** Multifocally within the vertebral canal and bordering the spinal cord there are hypointense foci (arrowheads), with the impression of an intradural-extramedullary mass lesion at T11-12 (arrow). Best seen on the HASTE sequence **(D)**, there is extensive attenuation of the subarachnoid space throughout the thoracic spine. There is no evidence of abnormal contrast enhancement **(E)**. Differential diagnoses included (granulomatous) meningitis, meningeal neoplasia and/or meningeal adhesions. **(H–J)** Autopsy was performed and yielded a diagnosis of extensive subdural and subarachnoid ossification with spinal cord compression.

A 7-year-old serval was presented with a several week history of ataxia and hind limb paresis unresponsive to medical treatment. The MRI examination of the thoracolumbar spine showed extensive attenuation of the subarachnoid space throughout the thoracic spine especially on the MR myelogram, with a suspected intradural extramedullary lesion at T11-12 (Figure 12). There was also evidence of multifocal degenerative intervertebral disc disease not causing spinal cord compression. Differential diagnoses included (granulomatous) meningitis, meningeal neoplasia and/or meningeal adhesions. Exploratory hemilaminectomy was performed and showed adhesions along the dorsal aspect of the spinal cord suspected to represent fibrosis and/or herniated disc material. Histopathologic examination of the samples was consistent with presence of adipose tissue and fibrosis. The animal died 4 days after the MRI, and autopsy yielded a diagnosis of extensive subdural and subarachnoid ossification with spinal cord compression.

#### Other Abnormalities

A 2-year-old snow leopard and an 8-year-old caracal had MRI evidence of spinal cord swelling/hyperintensity and diffuse attenuation of the subarachnoid space, respectively, suggesting possible meningomyelitis. CSF analysis in the snow leopard was normal, and the caracal had suspected, mild mixed pleocytosis. The snow leopard died 3.5 weeks following MRI, after developing systemic illness. Autopsy yielded a diagnosis of multifocal moderate Wallerian-type degeneration, and a primary axonopathy with myelin degeneration was suspected. The cause of death was attributed to adrenal insufficiency. The caracal died of presumptive septicemia 5 days after MRI. Autopsy yielded a diagnosis of mild to moderate Wallerian degeneration of the brain and spinal cord. There was no meningomyelitis in either animal, and the reason for the spinal cord and subarachnoid space changes suspected on MRI remains undetermined.

#### Normal MRI Studies

The MRI examination of the spine was considered unremarkable in seven felids.

A 0.7-year-old serval with a 3-month history of progressive paraparesis, weakness, ataxia and decreased postural reflexes in the pelvic legs had a normal MRI examination of the thoracolumbar spine. Electromyography (EMG) and nerve conduction velocity studies were performed. Muscular activity was normal, but peripheral nerve conduction recordings were decreased, consistent with a probable diagnosis of demyelinating/remyelinating polyneuropathy. The animal was treated medically, improved initially, and then had recurrent clinical signs 1.5 years after the MRI. Medical treatment was repeated and the patient improved, again.

A 6-year-old serval was presented for a 6-month history of self-mutilation of the tail and hyperesthesia. MRI of the lumbosacral spine was within normal limits, and a diagnosis of behavioral self-mutilation was made.

A 0.75-year-old cheetah was presented with progressive neurological deficits. MRI of the cervical spine (and brain) were normal. The CSF tap was consistent with hemorrhage, likely iatrogenic. Infectious disease testing was negative. Medical management was instituted at a referral institution. The animal was initially stable but then deteriorated and died <1 year following MRI. Autopsy findings were consistent with degenerative myelopathy.

A 10-year-old tiger became acutely non-ambulatory 2 weeks prior to presentation. CT of the thoracolumbar spine was performed and was within normal limits. The patient had minimal response to empiric treatment and was euthanized. A limited postmortem MRI examination of the cervical spine was performed and was unremarkable. On autopsy there was severe subacute myelomalacia of the lumbar spine (not included in the MRI field of view) attributed to fibrocartilaginous embolism although no emboli were detected in the examined sections.

In the remaining three animals the reason for neurologic deficits were found in the head/brain (*n* = 2) or remained undetermined (*n* = 1).

### Contrast Medium Administration

**Figure 13:**
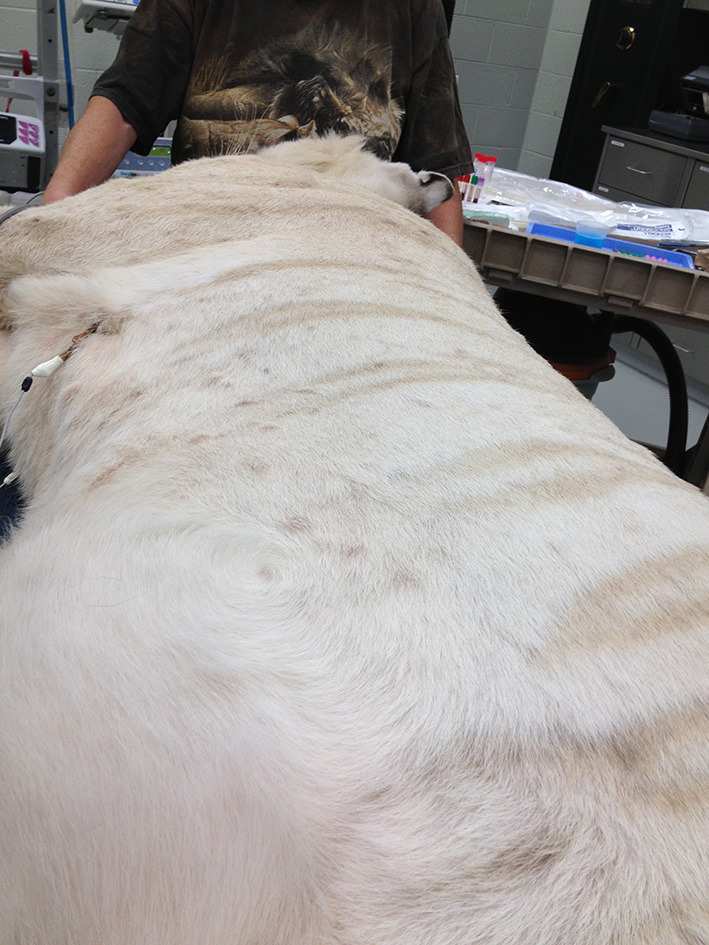
Urticaria in a white tiger noted at the end of the MRI examination and attributed to a reaction to contrast medium administration.

44 cats received a gadolinium-based MRI contrast agent (gadopentetate meglumine, Magnevist®, Bayer Schering Pharma; or gadodiamide, Omniscan®, GE Healthcase) intravenously at a dose of 0.1 mmol/kg and not exceeding 20 mL total volume. No adverse effects or other problems were noted in 43 cats. One patient, a white tiger, developed urticaria shortly after contrast medium administration (Figure 13), and an allergic adverse reaction was considered likely. The patient was managed medically (Benadryl, 1 mg/kg) and recovered uneventfully.

## Discussion

This manuscript presents the largest case series of captive non-domestic felids undergoing MRI of the CNS to date. Diagnostic quality MRI scans were acquired using a standard 1.0 or 1.5T MRI system and using modified standard brain and spine MRI protocols (21, 22). Intravenous contrast medium was administered routinely and was tolerated well in most animals with only one tiger exhibiting signs of an apparent allergic reaction. Gadolinium-based MRI contrast agents are generally considered safe for use in dogs and domestic cats with a low risk of serious adverse effect (23–25). Based on the results of this study they also have a low risk of adverse events in non-domestic cats.

The most common disorder was Chiari-like malformation which has previously been described in several feline species and has been associated with hypovitaminosis A in lions (8–10, 15, 16, 26–28). The imaging diagnosis is based on a combination of findings including crowding of the caudal fossa with or without foramen magnum herniation of the cerebellum, thickening of the occipital bone and osseous tentorium, and cervical syringomyelia. Several papers have reported numeric values (measurements of structures of the skull and brain and ratios) to allow for a less subjective diagnosis of this condition. One paper found the foramen magnum height to skull width ratio (FMH/SW) to be the most useful indicator (16), with normal lions having a mean ratio of 0.09 (range, 0.084-0.1) and 2 affected lions having measurements of 0.05 and 0.073, respectively. In the present study, values between both groups overlapped, and the mean between the affected and nonaffected groups were similar (0.06 vs. 0.069). This finding raises questions about the use of a single numeric measurement in the diagnosis of this complex condition.

A variety of inflammatory conditions was seen in this patient cohort. Generally, MRI findings with meningoencephalitis are variable. The study may be normal as in the bobcat diagnosed with neutrophilic meningoencephalitis, underlining the need for CSF analysis in conjunction with the MRI study if meningoencephalitis is of concern. If lesions are present on imaging, meningoencephalitis should be considered in cases of multifocal disease, an irregular lesion shape with ill-defined margins, and presence of meningeal contrast enhancement (29). These abnormalities were seen in a 1-year-old tiger with a postmortem diagnosis of lymphoplasmacytic meningoencephalitis. MRI abnormalities in one tiger with intracranial blastomycosis included asymmetric ventriculomegaly and contrast enhancement of the ventricular lining. While these changes resemble those seen in domestic cats with neurologic feline infectious peritonitis (30), they have also been reported in a small number of dogs with CNS blastomycosis (31), and at this point should not be considered pathognomonic for a specific infectious agent in large cats. The hemorrhagic tracts within the brain in one bobcat confirmed with autopsy are most consistent with parasite migration. The inciting organism was not identified. Intracranial parasite migration in dogs and cats has been reported with *Cuterebra, Capillaria, Taenia, Angiostrongylus* and *Baylisascaris* species (32–37). The identification of intracranial hemorrhage as seen in this patient requires a T2^*^-weighted GRE sequence or susceptibility weighted imaging, and inclusion of one of these sequences in the standard imaging protocol in large cats is recommended as it is for dogs and cats (38, 39).

Imaging findings in large felids with otitis media/interna were identical to those reported in domestic dogs and cats and included abnormal material within the tympanic bulla, bulla wall thickening, and alteration in normal fluid signal from the inner ear (40, 41).

The MRI diagnosis of ocular disease (corneal ulcer, keratitis and uveitis) in one patient was unexpected, and the decision to pursue advanced imaging in this patient may in part have been related to the inability to perform a thorough physical examination with the patient awake and attribution of ocular signs to CNS disease.

Pituitary abnormalities were noted in five animals and were considered incidental findings with the exception of a pituitary cystadenoma in a cougar with vision impairment. The finding of an empty sella in two patients is interesting as this has also previously been reported as an incidental finding on brain MRI in a tiger with suspected *Clostridium perfringens* neurotoxicity (12). Empty sella (herniation of the subarachnoid space into the sella turcica with absent or reduced size of the pituitary gland) in dogs may on occasion be associated with endocrinopathies but it is usually an incidental finding (42). Two animals had a nodular and cystic pituitary gland, respectively. Even though the clinical significance of these abnormalities ultimately remained undetermined, they were likely unrelated to the animals' presenting complaints. Incidental pituitary lesions in dogs are common and are seen in up to 13% of dogs with no presenting clinical signs of pituitary disease (43). The finding of presumably incidental pituitary lesions in approximately 10% of cases in this study should raise awareness of this abnormality when performing MRI studies in large felids.

An MRI diagnosis of severe bilaterally symmetric white matter disease was made postmortem in a 16-year-old snow leopard and confirmed with autopsy. Between 1994 and 2005, more than 70 adult captive felids (mostly cheetahs) were diagnosed with a novel leukoencephalomyelopathy of undetermined cause (11). An environmental husbandry-related neurotoxicity was suspected. Similar cases have sporadically been reported since (44, 45). A prominent feature of the disease in previously described cases was reactive astrocytosis which was not present in the patient in this report. Leukoencephalomyelopathy of unknown cause has also been reported in domestic cats, with vitamin A and B12 deficiency, feline parvovirus, feline leukemia virus, toxic and metabolic causes considered as possible etiologies (46).

Diffuse brain atrophy was the main finding in two geriatric cats. Even though there is clear evidence of age-associated brain pathology in domestic cats (47), reports on related imaging changes are scarce. Cerebral atrophy (decrease in both gray and white matter volume) with increased ventricular size and widened sulci is the most notable imaging finding (48) and was noted in our patients. In dogs and cats, imaging changes with “normal” brain aging and cognitive dysfunction overlap (49), and both etiologies were considered in these animals. The finding of gliosis and satellitosis (non-specific generalized cellular responses to CNS damage considered idiopathic) seen on autopsy in one of the animals is interesting and could indicate brain injury/disease unrelated to aging.

One patient was diagnosed with a skull fracture. The diagnosis was challenging, and the lesion was originally missed when the images were first evaluated. CT is generally considered the gold standard in the evaluation of acute traumatic head injury. MRI has been shown to have fairly high accuracy in fracture in identification in a canine and feline cadaver model (50). However, both the brain and cervical spine were imaged in this patient, and only a limited number of sequences and planes were acquired to minimize time under general anesthesia. In the future for similar cases where a traumatic etiology is considered and exact neurolocalization is not possible, CT of the area of interest may be given preference over MRI due to its superior bone imaging capabilities and the speed of image acquisition.

Neoplastic brain lesions (especially meningioma) are common in domestic felids (51) and were the most common etiologic category in a recent retrospective pathologic study of brain lesions in captive non-domestic felids (15). Only one animal included in this study had a neoplastic mass (pituitary cystadenoma) diagnosed on imaging. The reason for this discrepancy is unclear. Brain tumors occur most commonly in older animals (52, 53), and it is conceivable that euthanasia rather than expensive advanced imaging is chosen when neurologic signs develop in a geriatric felid. Another possible reason is that meningiomas may be microscopic (15) and could be missed on imaging. An intraventricular meningioma was identified on postmortem examination in one lion in this study which had not been present on the MRI examination 18 months prior to euthanasia and autopsy and likely developed in the meantime. Another surprising finding in this study was the lack of vascular anomalies which were common in the recent pathology study (15). The same animal diagnosed with a meningioma postmortem also had an infarct of the left caudate nucleus on autopsy that was not evident on the MRI study 18 months prior. The reason for the discrepancy in the diagnosis of vascular diseases between this imaging focused paper and the prior pathology study remains undetermined, however, it is possible that especially small vascular events did not result in MRI abnormalities but could have been present in some of the animals with a normal MRI examination.

The most common spinal disease in the current study was intervertebral disc disease. This is in line with several prior studies describing this condition as being common in non-domestic felids (4–6, 54).

Two animals were diagnosed with vertebral dysplasia resulting in spinal cord compression. This is surprising as this type of congenital vertebral anomaly is rather rare in animals. Spinal cord compression due to atlantal vertebral malformation has previously been reported in two African lions (3). It is conceivable that a decline in genetic diversity and inbreeding play a role in the higher than expected incidence of vertebral anomalies in captive non-domestic felids as has been previously documented for other species such as Scandinavian wolves (55).

The only patient with spinal neoplasia in this patient cohort was diagnosed with multiple myeloma, which is surprising [this animal was included in a prior case series on hypergammaglobulinemia and myeloma in 5 tigers (14)]. Spinal neoplasms in domestic cats are rare, and lymphoma is the most commonly reported tumor type (56, 57). Other previously reported tumor types in non-domestic felids include a thoracic vertebral chordoma, a vertebral osteosarcoma, and a benign peripheral nerve sheath tumor (45, 53, 58), but imaging findings for those cases were not reported. MRI findings in multiple myeloma in dogs overlap with other round cell tumors and include multifocal bone lesions centered on the medullary cavity with cortical sparing, with or without extension into the vertebral canal (56, 59), similar to changes seen in the patient of this report.

A presumptive diagnosis of an ischemic vascular accident was made in a 20-year-old clouded leopard. The location and appearance of the intramedullary lesion are consistent with an ischemic event. Even though the lesion resembled that of feline ischemic myelopathy (19, 20) in older domestic cats, the lesion was located in the dorsal rather than the ventral aspect of the spinal cord, and underlying arterial abnormalities described with that condition were not identified on autopsy in our patient. An ischemic event of a different etiology such as fibrocartilaginous embolism is therefore also considered. Cases of confirmed and suspected fibrocartilaginous embolism have previously been reported in non-domestic felids (13, 60).

Extensive subdural ossification resulting in spinal cord compression was diagnosed in one serval, an uncommon condition with only few similar cases reported in the veterinary literature (61, 62). The predominant MRI finding in this patient was extensive attenuation of the subarachnoid space on MR myelogram which is a nonspecific finding with a variety of possible causes including extradural compressive lesions, acute noncompressive spinal cord injury, spinal cord swelling, and/or infiltrative meningeal disease (63, 64). The hypointense nature of the material bordering the spinal cord and the impression of an intradural-extramedullary component of the lesion are consistent with presence of mineralized/ossified intradural/subdural material ultimately confirmed in surgery and autopsy.

The MRI examination was normal in several patients as expected with certain CNS diseases (e.g., idiopathic epilepsy, behavioral abnormalities, peripheral neuropathy, and degenerative myelopathy) or due to lesion location outside the image field of view or outside the CNS. While a normal MRI examination may be frustrating to the attending clinician when trying to identify the reason for an animal's presumed neurologic deficits, it is important to understand that even a normal study has value as it helps to rule out many diseases which would be expected to result in MRI abnormalities.

There are several limitations to this study. The information on clinical and neurologic examination findings in the medical records was limited in many cases, inherent to any retrospective study and complicated by the inherent difficulty in performing a clinical/neurologic exam in non-domestic felids. The decision which site to image was usually based on clinical signs reported by the caretakers and/or abnormalities observed by attending veterinarians. A decision to pursue brain MRI was based on abnormalities indicative of intracranial disease including seizures, head tilt, vision loss, and behavioral abnormalities. Imaging of the cervical spine was performed when gait abnormalities of all four legs were evident but there was no obvious evidence of concurrent abnormalities pertaining to the brain or cranial nerves. A decision to pursue MRI of the thoracolumbar/lumbosacral spine was based on abnormalities limited to the pelvic limbs or tail. Even though this resulted in imaging of the inappropriate area in some instances, and in some unnecessary MRI studies in animals with disease localization outside the CNS, the authors believe that this is representative of the veterinarians' and caretakers' situation when dealing with non-domestic felids. It is important to provide information on utility and limitations of performing MRI in the frame of the diagnostic workup for presumptive neurologic disease. Another limitation of this study is that a definitive diagnosis was not obtained in all animals to confirm the MRI diagnosis. Autopsy was performed in almost all animals that died during the evaluation period, and follow-up information was available for many others. However, there were a few cases for which either only an imaging diagnosis was obtained, or in which the diagnosis remained open. This is unfortunately not uncommon in retrospective studies. Another limitation is that the MRI protocols were not standardized. However, most studies included a minimum number of standard MRI sequences, and when performing an MRI examination in a clinical patient the protocol had to be tailored to the individual animal's situation. Sample MRI protocols are provided as appendices to this paper to aid veterinarians interested in performing an MRI study in a large non-domestic cat.

In summary, MRI is a valuable tool in the diagnostic workup of non-domestic felids with presumptive neurologic disease. This manuscript provides a summary of recommended MRI techniques and describes a variety of possible diagnoses including several previously unreported conditions.

## Data Availability Statement

The original contributions presented in the study are included in the article/Supplementary Material, further inquiries can be directed to the corresponding author.

## Author Contributions

SH devised the study, assisted with recording of the medical record data, interpreted the MRI studies, and prepared the manuscript. DW-H, KA, WT, AC, and ER recorded the medical record data, provided follow-up, and revised the manuscript. LC reviewed the histopathology for select cases and revised the manuscript. Final approval of the completed article was done by all authors.

## Conflict of Interest

The authors declare that the research was conducted in the absence of any commercial or financial relationships that could be construed as a potential conflict of interest.

## Publisher's Note

All claims expressed in this article are solely those of the authors and do not necessarily represent those of their affiliated organizations, or those of the publisher, the editors and the reviewers. Any product that may be evaluated in this article, or claim that may be made by its manufacturer, is not guaranteed or endorsed by the publisher.
