# Supplementary material for: Magnetic Resonance Imaging in 50 Captive Non-domestic Felids - Technique and Imaging Diagnoses
Source: Front Vet Sci. 2022 Feb 8;9:827870. doi: 10.3389/fvets.2022.827870 (PMC8861525; doi:10.3389/fvets.2022.827870)
Supplement: Supplementary file 2 [file Table_2.DOCX]

**Appendix 2 - MRI Protocols for Large Non-Domestic Felids**

**A) Sample MRI Protocol Brain at 1.0 T (105 kg tiger)**

| Sequence | Sequence type | TR (ms) | TE (ms) | TI (ms) | Flip Angle (°) | NEX | Slice thickness (mm) | Interslice gap (%) | Field of view (cm) | Acquisition matrix | Other |
| --- | --- | --- | --- | --- | --- | --- | --- | --- | --- | --- | --- |
| T2-W TSE sagittal | 2D | 4460 | 98 | N/A | 90° | 2 | 3 | 30 | 20 | 256 x 256 |  |
| T2-W TSE transverse | 2D | 4840 | 98 | N/A | 90° | 3 | 4 | 25 | 20 | 256 x 192 |  |
| T1-W SE transverse | 2D | 635 | 15 | N/A | 90° | 2 | 4 | 25 | 20 | 256 x 224 |  |
| T2-FLAIR transverse | 2D | 7500 | 102 | 2300 | 180° | 1 | 4 | 25 | 20 | 256 x 192 |  |
| T2*-W GRE transverse | 2D | 825 | 26 | N/A | 20° | 4 | 4 | 25 | 20 | 256 x 144 |  |
| T1-W SE transverse +C | 2D | 635 | 15 | N/A | 90° | 2 | 4 | 25 | 20 | 256 x 224 | +/- Fat Sat |
| T1-W SE sagittal +C | 2D | 580 | 15 | N/A | 90° | 1 | 3 | 25 | 20 | 256 x 256 | +/- Fat Sat |
| T1-W SE dorsal +C | 2D | 450 | 15 | N/A | 90° | 2 | 4 | 25 | 20 | 256 x 256 | +/- Fat Sat |

TSE = turbo spin echo; SE = spin echo; GRE = gradient recalled echo; T2-W = T2-weighted; T1-W = T1-weighted; T2-FLAIR = T2-weighted fluid attenuated inversion recovery; T2*-W = T2*-weighted; TR = time of repetition; TE = time of echo; TI = time of inversion; NEX = number of excitations; ms = milliseconds; mm = millimeter; cm = centimeter; +C = post IV contrast; Fat Sat = Chemical Fat Suppression

**B) Sample MRI Protocol Brain at 1.5 T (200 kg tiger)**

| Sequence | Sequence type | TR (ms) | TE (ms) | TI (ms) | Flip Angle (°) | NEX | Slice thickness (mm) | Interslice gap (%) | Field of view (cm) | Acquisition matrix | Other |
| --- | --- | --- | --- | --- | --- | --- | --- | --- | --- | --- | --- |
| T2-W TSE sagittal | 2D | 5000 | 105 | N/A | 90° | 2 | 4 | 17 | 32 | 384 x 288 |  |
| T2-W TSE transverse | 2D | 3900 | 107 | N/A | 90° | 2 | 4 | 25 | 28 | 384 x 288 |  |
| T1-W SE transverse | 2D | 360 | 14 | N/A | 90° | 2 | 4 | 25 | 28 | 256 x 192 |  |
| T2-FLAIR transverse | 2D | 5100 | 102 | 1820 | 180° | 2 | 4 | 25 | 28 | 256 x 192 |  |
| T2*-W GRE transverse | 2D | 850 | 13.3 | N/A | 20° | 1 | 4 | 25 | 28 | 256 x 230 |  |
| PD-W transverse | 2D | 2100 | 13 | N/A | 90° | 1 | 4 | 25 | 28 | 256 x 192 |  |
| DWI | 2D | 4000 | 114 | N/A | 90° | 3 | 5 | 30 | 28 | 192 x 192 | b-values 0, 500 and 1000 s/mm² |
| “SPACE” transverse | 3D | 1100 | 125 | N/A | variable | 2 | 2.5 | N/A | 28 | 320 x 317 |  |
| T1-W SE transverse +C | 2D | 400 | 14 | N/A | 90° | 2 | 4 | 25 | 28 | 256 x 192 | +/- Fat Sat |
| T1-W SE sagittal +C | 2D | 497 | 24 | N/A | 90° | 2 | 4 | 17 | 28 | 256 x 192 | +/- Fat Sat |
| T1-W SE dorsal+C | 2D | 572 | 14 | N/A | 90° | 2 | 4 | 25 | 28 | 256 x 192 | +/- Fat Sat |
| “VIBE” +C | 3D | 6.28 | 2.39 | N/A | 10° | 1 | 2.5 | N/A | 28 | 256 x 215 | Fat Sat |

TSE = turbo spin echo; SE = spin echo; GRE = gradient recalled echo; T2-W = T2-weighted; T1-W = T1-weighted; T2-FLAIR = T2-weighted fluid attenuated inversion recovery; PD-W = proton density-weighted; T2*-W = T2*-weighted; DWI = Diffusion weighted imaging; “SPACE” = “Sampling Perfection with Application optimized Contrasts using different flip angle Evolution”; “VIBE” = “Volume Interpolated Breathhold Examination”; TR = time of repetition; TE = time of echo; TI = time of inversion; NEX = number of excitations; ms = milliseconds; mm = millimeter; cm = centimeter; +C = post IV contrast; Fat Sat = Chemical Fat Suppression

**C) Sample MRI Protocol Spine at 1.0 T (131 kg lion)**

| Sequence | Sequence type | TR (ms) | TE (ms) | TI (ms) | Flip Angle (°) | NEX | Slice thickness (mm) | Interslice gap (%) | Field of view (cm) | Acquisition matrix | Other |
| --- | --- | --- | --- | --- | --- | --- | --- | --- | --- | --- | --- |
| STIR dorsal | 2D | 8760 | 16 | 150 | 180° | 2 | 4 | 24 | 50 | 320 x 168 |  |
| T2-W TSE sagittal | 2D | 4510 | 120 | N/A | 90° | 2 | 4 | 17 | 50 | 512 x 256 |  |
| T1-W TSE sagittal | 2D | 403 | 15 | N/A | 90° | 1 | 4 | 17 | 50 | 512 x 256 |  |
| STIR sagittal | 2D | 6350 | 17 | 150 | 180° | 1 | 4 | 10 | 50 | 320 x 224 |  |
| “HASTE” sagittal | 3D | 7000 | 277 | N/A | 150° | 1 | 1.5 | N/A | 50 | 256 x 256 |  |
| T2-W TSE transverse | 2D | 3520 | 98 | N/A | 90° | 3 | 6 | 20 | 25 | 256 x 192 |  |
| T1-W SE transverse | 2D | 518 | 20 | N/A | 90° | 2 | 6 | 20 | 25 | 256 x 192 |  |
| T2*-W GRE transverse | 2D | 610 | 26 | N/A | 20° | 1 | 6 | 20 | 25 | 256 x 256 |  |
| T1-W SE sagittal +C | 2D | 574 | 15 | N/A | 90° | 1 | 4 | 17 | 50 | 512 x 256 | + Fat Sat |
| T1-W SE transverse +C | 2D | 932 | 20 | N/A | 90° | 1 | 6 | 20 | 25 | 384 x 288 | + Fat Sat |

STIR = Short tau inversion recovery; TSE = turbo spin echo; SE = spin echo; GRE = gradient recalled echo; T2-W = T2-weighted; T1-W = T1-weighted; HASTE = Half-Fourier Acquisition Single-shot Turbo spin Echo imaging (MR myelogram); TR = time of repetition; TE = time of echo; TI = time of inversion; NEX = number of excitations; ms = milliseconds; mm = millimeter; cm = centimeter; +C post IV contrast; Fat Sat = Chemical Fat Suppression

**D) Sample MRI Protocol Spine at 1.5 T (200 kg tiger)**

| Sequence | Sequence type | TR (ms) | TE (ms) | TI (ms) | Flip Angle (°) | NEX | Slice thickness (mm) | Interslice gap (%) | Field of view (cm) | Acquisition matrix | Other |
| --- | --- | --- | --- | --- | --- | --- | --- | --- | --- | --- | --- |
| STIR dorsal | 2D | 4000 | 37 | 160 | 180° | 2 | 3.5 | 10 | 38 | 256 x 174 |  |
| T2-W TSE sagittal | 2D | 3200 | 100 | N/A | 90° | 2 | 3 | 10 | 38 | 256 x 205 | +/- Composing to extend field of view |
| T1-W TSE sagittal | 2D | 333 | 13 | N/A | 90° | 2 | 3 | 10 | 38 | 384 x 269 |  |
| STIR sagittal | 2D | 4000 | 37 | 160 | 180° | 2 | 3 | 10 | 38 | 256 x 192 |  |
| “HASTE” sagittal | 3D | 7000 | 217 | N/A | 150° | 1 | 0.9 | N/A | 38 | 256 x 256 |  |
| T2*-W GRE sagittal | 2D | 850 | 26 | N/A | 20° | 1 | 3 | 10 | 38 | 256 x 192 |  |
| “SPACE” transverse | 3D | 1350 | 121 | N/A | variable | 2 | 3 | N/A | 24 | 320 x 317 |  |
| T2-W TSE transverse | 2D | 3790 | 95 | N/A | 90° | 1 | 6 | 20 | 25 | 320 x 240 |  |
| T1-W SE transverse | 2D | 501 | 13 | N/A | 90° | 1 | 6 | 20 | 25 | 320 x 240 |  |
| T1-W SE sagittal +C | 2D | 475 | 13 | N/A | 90° | 1 | 3 | 10 | 38 | 384 x 269 | + Fat Sat |
| T1-W SE transverse +C | 2D | 597 | 13 | N/A | 90° | 1 | 6 | 20 | 25 | 320 x 240 | + Fat Sat |

STIR = Short tau inversion recovery; TSE = turbo spin echo; SE = spin echo; GRE = gradient recalled echo; T2-W = T2-weighted; T1-W = T1-weighted; HASTE = Half-Fourier Acquisition Single-shot Turbo spin Echo imaging (MR myelogram); “SPACE” = “Sampling Perfection with Application optimized Contrasts using different flip angle Evolution”; TR = time of repetition; TE = time of echo; TI = time of inversion; NEX = number of excitations; ms = milliseconds; mm = millimeter; cm = centimeter; +C post IV contrast; Fat Sat = Chemical Fat Suppression
